# Characteristics of patients with non-cancer pain and long-term prescription opioid use who have used medical versus recreational marijuana

**DOI:** 10.1186/s42238-024-00218-y

**Published:** 2024-02-22

**Authors:** Whitney M. Davidson, Anika Mahavni, Timothy Chrusciel, Joanne Salas, Lisa R. Miller-Matero, Mark D. Sullivan, Celeste Zabel, Patrick J. Lustman, Brian K. Ahmedani, Jeffrey F. Scherrer

**Affiliations:** 1 Department of Family and Community Medicine, Saint Louis University School of Medicine, 1008 S. Spring, SLUCare Academic Pavilion, 3rd Floor, St. Louis, MO 63110 USA; 2 Department of Psychiatry and Behavioral Neuroscience, Saint Louis University School of Medicine, 1438 South Grand Blvd., St. Louis, MO 63104 USA; 3 Advanced HEAlth Data (AHEAD) Research Institute, Saint Louis University School of Medicine, 3545 Lafayette Ave, 4th Floor, St. Louis, MO 63104 USA; 4 Center for Health Policy and Health Services Research and Behavioral Health Services, Henry Ford Health, One Ford Place, Detroit, MI 48202 USA; 5 Department of Psychiatry and Behavioral Science, University of Washington School of Medicine, 1959 NE Pacific Street, Seattle, WA 98195 USA; 6 Department of Health and Clinical Outcomes Research, Saint Louis University School of Medicine, 3545 Lafayette Ave, 4th Floor, St. Louis, MO 63104 USA; 7 Department of Psychiatry, Washington University School of Medicine, 4320 Forest Park Blvd, Suite 301, St. Louis, MO 63108 USA

**Keywords:** Pain, Opioid, Psychiatry, Cohort, Epidemiology

## Abstract

**Objective:**

Marijuana use is increasingly common among patients with chronic non-cancer pain (CNCP) and long-term opioid therapy (LTOT). We determined if lifetime recreational and medical marijuana use were associated with more frequent and higher dose prescription opioid use.

**Design:**

Cross-sectional

**Subjects:**

Eligible patients (*n*=1,037), who had a new period of prescription opioid use lasting 30-90 days, were recruited from two midwestern health care systems to a study of long-term prescription opioid use and mental health outcomes. The present cross-sectional analyses uses baseline data from this on-going cohort study.

**Methods:**

Primary exposures were participant reported lifetime recreational and medical marijuana use versus no lifetime marijuana use. Prescription opioid characteristics included daily versus non-daily opioid use and ≥50 morphine milligram equivalent (MME) dose per day vs. <50 MME. Multivariate, logistic regression models estimated the association between lifetime recreational and medical marijuana use vs. no use and odds of daily and higher dose prescription opioid use, before and after adjusting for confounding.

**Results:**

The sample was an average of 54.9 (SD±11.3) years of age, 57.3% identified as female gender, 75.2% identified as White, and 22.5% identified as Black race. Among all participants, 44.4% were never marijuana users, 21.3% were recreational only, 7.7% medical only and 26.6% were both recreational and medical marijuana users. After controlling for all confounders, lifetime recreational marijuana use, as compared to no use, was significantly associated with increased odds of daily prescription opioid use (OR=1.61; 95%CI:1.02-2.54). There was no association between lifetime recreational or medical marijuana use and daily opioid dose.

**Conclusion:**

Lifetime medical marijuana use is not linked to current opioid dose, but lifetime recreational use is associated with more than a 60% odds of being a daily prescription opioid user. Screening for lifetime recreational marijuana use may identify patients with chronic pain who are vulnerable to daily opioid use which increases risk for adverse opioid outcomes. Prospective data is needed to determine how marijuana use influences the course of LTOT and vice versa.

## Introduction

The United States has experienced a rapid increase in access to medical and recreational marijuana. Currently, 38 states permit medical marijuana, and recreational use of marijuana is legal in 23 states (https://disa.com/marijuana-legality-by-state n.d.). Though chronic pain is commonly treated with prescription opioids, patients are increasingly reporting medical marijuana use for pain management (Kosiba et al. 2019; Park and Wu 2017; Wadsworth et al. 2022).

At the individual level, there is evidence indicating medical marijuana use is associated with decreased opioid use, in some instances up to a 64% decrease (Boehnke et al. 2016; Wen et al. 2021). A recent study indicates medical marijuana may have the greatest impact on reducing opioid dose among those receiving higher daily morphine milligram equivalent (MME) doses (Nguyen et al. 2023). Recent survey data revealed that approximately 30% of dual medical marijuana and prescription opioid users perceived marijuana as a means to reduce and eventually stop opioid use (Bobitt et al. 2023). In fact, up to 76% of medical marijuana users report reducing opioid use after starting medical marijuana (Piper et al. 2017). Marijuana may have opioid sparing effects, but the certainty of existing evidence is low (Noori et al. 2021).

It has been observed that opioid related mortality was lower in areas with a higher prevalence of medical dispensaries (Hsu and Kovacs 2021). Although these studies suggest medical marijuana may be linked to reduced opioid consumption, patients with chronic pain who use marijuana for analgesia experienced worsened anxiety symptoms and higher pain intensity (Campbell et al. 2018). One study reported chronic pain patients who used marijuana experienced subsequent nonmedical prescription opioid use/opioid use disorder (Olfson et al. 2018). In addition, there is evidence that marijuana does not reduce pain severity among persons who use both or either opioids or marijuana (Campbell et al. 2018). In fact, one of the most common reasons for stopping the use of marijuana is insufficient analgesia (Boehnke et al. 2021).

As the legalization of recreational marijuana increases in the United States, patterns of consumption may change in individuals with chronic pain. In assessing the use of medical marijuana for recreational purposes, one study indicated that 55.5% of patients who use medical marijuana legally, also use marijuana for recreation (Morean and Lederman 2019). Patients who use marijuana for non-cancer pain may differ from those who use marijuana for recreational purposes and these patients may differ from those who use marijuana for both pain relief and recreation. Given previous findings that suggest opioid use may decrease with medical marijuana use, and evidence that marijuana is not an effective analgesic, further research is warranted to understand the association between lifetime patterns of marijuana consumption and odds of daily high dose opioid use. Additionally, given the high prevalence of comorbid pain and mental illness, we sought to determine if associations between marijuana and opioid use were independent of comorbid mental health conditions and substance use disorder (SUD).

This study uses data from the Prescription Opioids and Depression Pathways Cohort Study, henceforth termed “the Pathways Study.” In the present cross-sectional design we sought to 1) determine if the associations between lifetime marijuana use and current daily vs. non-daily opioid use vary by recreational, medical or use of both recreational and medical marijuana; and 2) determine if lifetime recreational and medical marijuana use vary in their association with odds of receiving higher opioid dose; and 3) determine if associations between marijuana and opioid outcomes are independent of confounding from comorbid mental illness and SUD.

## Material and methods

### Subjects

Eligible patients were selected from the electronic health records (EHRs) of Saint Louis University (SLU) in St. Louis, MO, and Henry Ford Health (HFH) in Detroit, MI based on predetermined inclusion and exclusion criteria. Patients were eligible if they were 18-70 years of age, starting a new period of 30-to-90-day prescription opioid use (no opioid use in the prior 3 months), and free of cancer and HIV. These criteria were verified via both the EHR and a screening questionnaire. The EHR algorithm identified eligible subjects on a weekly basis by searching pharmacy records of patients and selecting those who had received sufficient opioid prescriptions to be 30-to-90-day users and then limiting this group to those who did not have an opioid prescription in the 3 months prior to this new episode of prescription opioid use. Recruitment packets were sent to eligible subjects who were encouraged to use the internet to complete the survey or to contact a study research assistant to complete the interview by phone. Subjects were given 5 weeks to complete the survey. Persons who did not participate after receiving the recruitment packet were contacted by research staff via telephone. Up to 10 phone calls over 5 weeks were made to recruit participants. A total of 1,047 patients completed the baseline assessment between November 2019 and November 2022. Follow-up data collection and data cleaning is on-going which is why we used baseline measures. Additional details for study recruitment and retention have been described elsewhere (Scherrer et al. 2020).

### Variables

#### Exposure

The primary exposure of this study was type of marijuana use (i.e., medical versus recreational versus both). Reason for marijuana use was queried using the following questions. Participants were asked, 1) “Have you ever used marijuana to feel good or high?” and 2) “Have you ever used marijuana to control pain?” Those reporting only use to feel good or high were classified as recreational users and those who reported use only for pain were classified as medical marijuana users. From these questions we created a four-level marijuana exposure: 1) participants who used both lifetime medical and recreational, 2) those who were lifetime recreational only, 3) lifetime medical only, or 4) were lifetime non-users.

#### Outcomes

Participants reported the characteristics of their opioid prescription by reporting on the following items: 1) type of opioid in the past 90 days (e.g., prescriptions for codeine, dihydrocodeine, fentanyl, hydrocodone, etc.); 2) opioid daily dose, using number of milligrams per pill/dose and number of pills taken per day, which was converted to morphine milligram equivalent dose (MME). MME dose was dichotomized as < 50 morphine milligram equivalent (MME) dose per day vs. ≥50 MME per day; and 3) days per week opioid was used which was categorized into daily and non-daily use.

##### Patient characteristics/Covariates

Demographic measures included age, gender (man, woman), race (white, black, other), and marital status (married/live with partner, never married, widowed/divorced/separated). Pain measures were evaluated using the Brief Pain Inventory (BPI) which assessed pain severity and interference (Cleeland 1991; Keller et al. 2004). A pain severity score was calculated as the average of the following 4 items on a scale from “0=no pain” to “10=pain as bad as you can imagine”: worst in last 30 days, least in last 30 days, pain on average, and current pain. Pain interference was calculated as the average score from seven questions that asked participants to rate on a “0=does not interfere” to “10=completely interferes” scale about whether pain has interfered with general activity, mood, walking ability, normal work, relationships with other people, sleep, and enjoyment of life in the last 30 days.

The Prescription Opioids Difficulty Scale (PODS) was administered to obtain measures on psychosocial problems patients attribute to opioid use and concerns about opioid use. Higher scores indicate greater problems with opioids, and scores ≥ 16 were considered high (Banta-Green et al. 2010). The PODS is “focused on the patients’ perspective,” and it distinguishes harms related to psychosocial problems and concerns about use that are distinct from opioid use disorder (Banta-Green et al. 2009; Sullivan 2010). We also administered the Current Opioid Misuse Measure (COMM) (Butler et al. 2010) because it includes items to define less severe misuse. The PODS and COMM were not collinear.

Mental health measures included depression, anxiety, posttraumatic stress disorder (PTSD) anhedonia and substance use disorder. We used a computerized version of the Semi-Structured Assessment for the Genetics of Alcoholism (SSAGA-II) (Bucholz et al. 1994) to obtain DSM-IV depression and any type of substance use disorder (SUD) diagnoses. Anxiety was measured with the Generalized Anxiety Disorder scale-7 (GAD-7) (Spitzer et al. 2006; Williams 2014). Higher GAD-7 scores indicate worse anxiety and a score ≥ 15 indicates probable GAD (Kessler et al. 2001). PTSD was assessed with the Primary Care-PTSD-5 (PC-PTSD-5) (Prins et al. 2016), where a score of at least three was coded as probable PTSD. The Snaith-Hamilton Pleasure Scale (SHAPS) was used to measure anhedonia (Snaith et al. 1995) with higher scores indicating more severe anhedonia and a score ≥ 3 indicating high anhedonia (Franken et al. 2007). Current smoking was classified as smoking cigarettes every day or some days, versus past (smoked at least 100 cigarettes in entire life but does not currently smoke) and never smoked. We controlled for vital exhaustion which is characterized by loss of energy, demoralization, and irritability (Appels and Mulder 1989; Meesters and Appels 1996). We adjusted for ability to participate in social roles via the PROMIS SR (Hahn et al. 2010) and high social support via the PROMIS ES (http://www.healthmeasures.net/search-view-measures?task=Search.search. n.d.).

### Analytic Approach

All analyses were weighted using stabilized inverse probability of participation weights so that results could be generalized to all eligible patients with non-cancer pain and a new period of 30-90 days of opioid use. Propensity scores (PS) for participation in the Pathways Study were calculated using a binary logistic regression model assessing the conditional participation based on age, race, gender, and electronic health record variables (arthritis, back/neck pain, muscle pain, fibromyalgia, chronic pain, neuropathy, headache, any substance use disorder, depression, and anxiety). Stabilized weights were calculated by multiplying the inverse of the propensity score by the observation participation rate. Stabilized weights reduce bias associated with extreme rates and preserve original sample size in analyses (Curtis et al. 2007; Rosenbaum and Rubin 1983; Xu et al. 2010). Assessment of balance for included model variables was calculated using the standardized mean difference percent (SMD%). All variables balanced between those participating and not participating in the study as all SMD% were <10% (Austin and Stuart 2015).

After deleting four cases with missing data regarding marijuana use and applying the stabilized weights, there were 1,037 participants available for study analyses. Bivariate associations between covariates and opioid use variables were assessed using Chi-square tests for categorical variables and analysis of variance (ANOVA) for continuous variables. Logistic regression models were computed in a stepwise fashion. First, a crude model estimated the association between type of marijuana use and odds of daily versus non-daily opioid use. Logistic regression model building proceeded by first adding demographics to the crude model, then adding mental illness and SUD variables and last, the fully adjusted model added additional control for pain and opioid use characteristics. This process was repeated for modeling receipt of ≥50 MME versus <50 MME dose per day. Measures of association were expressed as odds ratios and 95% confidence intervals. No significant multicollinearity was detected between covariates based on tolerance <0.10 or Variance Inflation Factor >10. Alpha of 0.05 was used for all statistical tests. SAS v9.4 (Cary, NC) was used for all analyses.

## Results

Overall, the cohort was 54.9 (SD±11.3) years of age, 57.3% female gender, 75.2% White and 22.5% Black race. Among all participants, 44.4% were never marijuana users, 21.3% were recreational only, 7.7% medical only and 26.6% were both recreational and medical marijuana users. Recreational only and use of both recreational and medical cannabis were more prevalent among respondents with daily opioid use versus non-daily use (*p*=0.033). Younger age was more common in daily opioid users (*p*=0.0489) as was identifying as White (*p*=0.0225) (Table 1). High pain severity (*p*=0.023) and high pain interference (*p*<0.0001) were significantly more common among daily opioid users. The average number of pain sites was significantly (*p*=0.0029) greater among daily users. Lifetime depression (*p*=0.0259), positive PODS score (*p*<0.0001), any SUD (*p*<0.0001) and current smoking (*p*<0.0001) were significantly associated with daily opioid use. Ability to participate in social activities (*p*=0.0231) was significantly less common among daily opioid users. Male gender (*p*=0.0258), high pain interference (*p*=0.0038), anxiety (*p*=0.015) and positive PODS (*p*<0.0001) were more prevalent among patients receiving ≥50 MME. High emotional support was more prevalent among respondents who received <50 MME (Table 1).

**Table 1:** Sample characteristics (%, *n*) overall and by daily opioid use and by morphine milligram equivalent dose (MME)

	Overall	Daily Opioid			MME >= 50		
	(*n*=1,037)	Yes (*n*=686)	No (*n*=327)	*p*-value	Yes (*n*=159)	No (*n*=759)	*p*-value
Marijuana Use							
No Marijuana	44.4% (461)	41.5% (285)	51.2% (167)	0.0330	47.1% (75)	44.3% (336)	0.6553
Recreational Only	21.3% (221)	22.6% (155)	17.7% (58)		21.1% (34)	20.4% (155)	
Medical only	7.7% (80)	8.0% (55)	7.1% (23)		5.7% (9)	8.6% (65)	
Both	26.6% (276)	27.9% (191)	24.1% (79)		26.1% (42)	26.7% (203)	
Age (mean, SD)	54.9 (11.3)	54.4 (11.3)	55.9 (11.3)	0.0489	54.2 (10.8)	55.1 (11.3)	0.3625
Gender							
Man	42.7% (442)	44.6% (305)	38.3% (125)	0.0565	50.8% (81)	41.2% (312)	0.0258
Woman	57.3% (594)	55.4% (379)	61.7% (201)		49.2% (78)	58.8% (446)	
Race							
White	75.2% (758)	77.4% (516)	70.0% (222)	0.0225	80.4% (126)	74.9% (551)	0.1718
Black	22.5% (227)	20.1% (134)	27.9% (89)		16.2% (25)	22.8% (168)	
Other	2.3% (23)	2.5% (17)	2.1% (7)		3.3% (5)	2.4% (18)	
Marital Status							
Married/live with partner	53.0% (543)	52.8% (357)	54.3% (176)	0.6961	53.8% (84)	53.6% (403)	0.9880
Widow/Div/Sep	30.1% (309)	30.9% (209)	28.4% (92)		29.3% (46)	29.9% (224)	0.9880
Never married	16.9% (174)	16.3% (110)	17.4% (56)		16.9% (26)	16.5% (124)	0.9880
High pain severity	28.7% (298)	31.4% (216)	24.5% (80)	0.0230	29% (46)	29.1% (220)	0.9812
High pain interference	33.0% (342)	37.2% (255)	24.5% (80)	<.0001	43.2% (69)	31.3% (237)	0.0038
# Pain Sites (mean, SD)	5.9 (3.7)	6.2 (3.7)	5.5 (3.5)	0.0029	5.8 (3.8)	6.0 (3.6)	0.5320
Lifetime Depression	31.7% (329)	33.9% (233)	27% (88)	0.0259	32.9% (52)	31.9% (242)	0.7934
Anxiety^a^	19.4% (201)	20.5% (140)	17.6% (57)	0.2763	25.8% (41)	17.5% (132)	0.0150
PTSD^b^	15.7% (159)	16.5% (111)	14.2% (45)	0.3676	16% (25)	15.2% (112)	0.7968
Anhedonia	28.8% (294)	29% (195)	29.1% (93)	0.9590	27.5% (43)	29.5% (219)	0.6106
COMM^c^	30.7% (314)	32.7% (222)	28.6% (91)	0.1966	35.5% (56)	30.7% (229)	0.2406
PODS^d^	17.0% (174)	21.6% (146)	8.6% (28)	<.0001	33.4% (53)	14.8% (111)	<.0001
Vital Exhaustion	18.0% (185)	18.9% (129)	16.9% (55)	0.4435	21.7% (34)	17.2% (130)	0.1753
High Emotional Support^e^	53.6% (551)	52.6% (358)	54.6% (176)	0.5389	45.8% (73)	54.8% (412)	0.0384
High Social Role Functioning^f^	12.0% (122)	10.1% (68)	15% (48)	0.0231	10.4% (16)	12.2% (91)	0.5401
Any SUD^g^	12.6% (131)	16.1% (110)	5.5% (18)	<.0001	17.0% (27)	12.5% (95)	0.1263
Current Smoker	26.6% (275)	30.7% (209)	18.4% (60)	<.0001	28.1% (45)	26.3% (198)	0.6335

^a^Anxiety – Generalized Anxiety Disorder

^b^PTSD – Posttraumatic Stress Disorder

^c^COMM – Current Opioid Misuse Measure

^d^PODS - Prescribed Opioid Difficulties Scale

^e^High Emotional Support – PROMIS ES

^f^High Social Role Functioning – PROMIS SR

^g^SUD – Substance Use Disorder

As shown in Table 2, prior to adjusting for covariates we observed a significant association between recreational marijuana use, as compared to no use, and odds of daily prescription opioid use (OR=1.58; 95%CI:1.11-2.26). Users of both recreational and medical marijuana, as compared to no marijuana use, were significantly more likely to be daily opioid users (OR=1.43; 95%CI:1.03-1.98). After adjusting for demographics in model 2, the associations between marijuana use and daily opioid use remained largely unchanged. After controlling for mental illness and SUD in model 3, there were no significant associations between any type of marijuana use and daily prescription opioid use. However, after controlling for pain measures, opioid misuse, vital exhaustion, and emotional and social support in the fully adjusted model 4, the association between recreational marijuana only and daily opioid use remained significant (OR=1.61; 95%CI:1.02-2.54). Results from the full model also indicated an inverse association between race and daily opioid use (OR=0.56; 95%CI:0.38-0.84). Any SUD, current smoking, high pain interference, number of pain sites and a positive PODS score were all significantly and positively associated with daily opioid use.

**Table 2:** Logistic regression models for the association between recreation, medical or both types of marijuana use vs. no marijuana use and odds of daily prescription opioid use

	Model 1: Unadjusted OR (95%CI)	Model 2: Demographics OR (95%CI)	Model 3: MH and SUD OR (950025CI)	Model 4: Full Adjusted Model OR (95%CI)
Marijuana Use				
No Marijuana	1.0	1.0	1.0	1.0
Recreational Only	**1.58 (1.11-2.26)**	**1.70 (1.13-2.55)**	1.34 (0.87-2.07)	**1.61 (1.02-2.54)**
Medical only	1.39 (0.83-2.35)	1.11 (0.64-1.93)	1.09 (0.62-1.92)	0.98 (0.54-1.77)
Both	**1.43 (1.03-1.98)**	1.25 (0.88-1.80)	1.02 (0.69-1.52)	1.08 (0.71-1.64)
Age		**0.98 (0.97-1.00)**	0.99 (0.97-1.00)	0.98 (0.97-1.00)
Male gender		1.32 (0.98-1.79)	1.27 (0.93-1.75)	1.26 (0.90-1.77)
Race				
White		1.0	1.0	1.0
Black		**0.68 (0.48-0.97)**	0.70 (0.48-1.01)	**0.56 (0.38-0.84)**
Other		1.32 (0.49-3.56)	1.25 (0.42-3.73)	1.05 (0.34-3.26)
Marital Status				
Married/ live with partner		1.0	1.0	1.0
Widow/Div/Sep		1.14 (0.81-1.60)	1.08 (0.76-1.54)	1.11 (0.76-1.61)
Never married		0.96 (0.62-1.48)	0.90 (0.58-1.42)	0.88 (0.55-1.41)
Lifetime Depression			1.07 (0.73-1.57)	0.92 (0.61-1.40)
Anxiety^a^			0.90 (0.56-1.42)	0.75 (0.45-1.27)
PTSD^b^			0.88 (0.54-1.43)	0.85 (0.50-1.44)
Anhedonia			0.93 (0.65-1.34)	0.86 (0.57-1.29)
	Recreational vs. no marijuana OR (95%CI)	Medical vs. no marijuana OR (95%CI)	Both medical and recreational vs. no marijuana OR (95%CI)	Model 4: Full Adjusted Model OR (95%CI)
Any SUD^c^			**3.13 (1.70-5.76)**	**3.07 (1.62-5.82)**
Current Smoker			**1.75 (1.19-2.58)**	**1.63 (1.09-2.45)**
High pain severity				1.23 (0.82-1.84)
High pain interference				**1.67 (1.12-2.48)**
# Pain Sites				**1.05 (1.00-1.10)**
COMM^d^				0.88 (0.58-1.32)
PODS^e^				**2.57 (1.56-4.24)**
Vital Exhaustion				0.69 (0.40-1.19)
High Emotional Support^f^				0.84 (0.60-1.18)
High Social Role Functioning^g^				0.72 (0.44-1.17)

Bold text indicates statistically significant (*p*<0.05) odds ratios

^a^Anxiety – Generalized Anxiety Disorder

^b^PTSD – Posttraumatic Stress Disorder

^c^SUD – Substance Use Disorder

^d^COMM – Current Opioid Misuse Measure

^e^PODS - Prescribed Opioid Difficulties Scale

^f^High Emotional Support – PROMIS ES

^g^High Social Role Functioning – PROMIS SR

As shown in Table 3, there was no association between type of marijuana use and odds of receiving ≥ 50 MME dose. This did not change in fully adjusted models. Results from the full model revealed that Black race was inversely associated with odds of receiving ≥ 50 MME dose (OR=0.58; 95%CI:0.34-0.99). High pain interference (OR=1.82; 95%CI:1.18-2.81) and positive PODS (OR=3.08; 95%CI:1.96-4.84) were significantly associated with increased odds of receiving ≥ 50 MME.

**Table 3:** Logistic regression models for the association between recreation, medical or both types of marijuana use vs. no marijuana use and odds of receiving ≥50 MME prescription opioid dose

	Model 1: Unadjusted OR (95%CI)	Model 2: Demographics OR (95%CI)	Model 3: MH and SUD OR (95%CI)	Model 4: Full Adjusted Model OR (95%CI)
Marijuana Use				
No Marijuana	1.0	1.0	1.0	1.0
Recreational Only	0.97 (0.62-1.53)	0.90 (0.56-1.43)	0.88 (0.54-1.45)	1.00 (0.59-1.70)
Medical only	0.63 (0.30-1.31)	0.54 (0.25-1.17)	0.51 (0.23-1.12)	0.52 (0.23-1.16)
Both	0.92 (0.60-1.39)	0.81 (0.52-1.26)	0.76 (0.48-1.22)	0.79 (0.48-1.31)
Age		0.99 (0.97-1.00)	0.99 (0.98-1.01)	0.99 (0.97-1.01)
Male gender		1.42 (0.99-2.04)	**1.46 (1.01-2.12)**	1.38 (0.93-2.03)
Race				
White		1.0	1.0	1.0
Black		**0.57 (0.35-0.93)**	**0.59 (0.36-0.97)**	**0.58 (0.34-0.99)**
Other		1.38 (0.50-3.80)	1.65 (0.58-4.69)	1.42 (0.47-4.27)
Marital Status				
Married/ live with partner		1.0	1.0	1.0
Widow/Div/Sep		1.12 (0.75-1.69)	1.05 (0.69-1.59)	1.14 (0.73-1.78)
Never married		1.11 (0.66-1.87)	1.07 (0.63-1.82)	1.20 (0.68-2.09)
Lifetime Depression			0.96 (0.61-1.49)	0.72 (0.44-1.18)
Anxiety^a^			**2.04 (1.22-3.41)**	1.48 (0.83-2.62)
PTSD^b^			0.97 (0.56-1.70)	0.87 (0.48-1.59)
Anhedonia			0.73 (0.47-1.12)	0.72 (0.44-1.18)
	Recreational vs. no marijuana OR (95%CI)	Medical vs. no marijuana OR (95%CI)	Both medical and recreational vs. no marijuana OR (95%CI)	Model 4: Full Adjusted Model OR (95%CI)
Any SUD^c^			1.24 (0.73-2.10)	1.10 (0.63-1.92)
Current Smoker			0.99 (0.65-1.52)	1.00 (0.63-1.57)
High pain severity				0.95 (0.60-1.51)
High pain interference				**1.82 (1.18-2.81)**
# Pain Sites				0.96 (0.91-1.02)
COMM^d^				0.88 (0.55-1.41)
PODS^e^				**3.08 (1.96-4.84)**
Vital Exhaustion				1.25 (0.68-2.30)
High Emotional Support^f^				0.70 (0.46-1.04)
High Social Role Functioning^g^				1.10 (0.59-2.05)

Bold text indicates statistically significant (*p*<0.05) odds ratios

^a^Anxiety – Generalized Anxiety Disorder

^b^PTSD – Posttraumatic Stress Disorder

^c^SUD – Substance Use Disorder

^d^COMM – Current Opioid Misuse Measure

^e^PODS - Prescribed Opioid Difficulties Scale

^f^High Emotional Support – PROMIS ES

^g^High Social Role Functioning – PROMIS SR

## Discussion

In a large cohort of patients with chronic non-cancer pain who were starting a new period of prescription opioid use lasting at least 30 days, we observed lifetime *recreational* marijuana users, as compared to never users, had a 61% increased odds of current daily prescription opioid use. There was no association between lifetime *medical* marijuana use alone or in combination with recreational use and odds of being a daily opioid user. There were no associations between recreational or medical marijuana use and odds of higher opioid dose.

These results were obtained after controlling for a large number of potential confounding factors including demographics, pain interference, number of pain sites, lifetime depression, vital exhaustion, any SUD, and current smoking. Importantly, the relationship between recreational marijuana use and daily opioid use was independent of opioid misuse and other forms of SUD which offers some evidence that a general orientation to substance use problems does not explain lifetime co-use of recreational marijuana and frequent prescription opioid consumption.

While there is a large literature on cannabis use and pain and prescription opioid outcomes, to our knowledge, there are few existing studies that have examined whether the associations between lifetime recreational versus lifetime medical marijuana use differ in association with daily, higher dose prescription opioid use. Our results are not consistent with evidence that prescription opioid use is more than twice as common among medical as compared to recreational marijuana users (Goulet-Stock et al. 2017). However direct comparison is difficult because the latter study did not recruit patients receiving opioids for non-cancer pain. Results from the 4-year prospective Pain and Opioids IN Treatment (POINT) study, found no evidence that marijuana reduced prescription opioid use (Campbell et al. 2018). Given the inconsistencies in existing literature, additional prospective cohort studies are needed. Differences in sample characteristics and variation in approach to measuring marijuana and opioid use may contribute to these incongruous findings. Future work should be careful to measure marijuana use using standardized instruments.

Whether marijuana legalization reduces high dose, LTOT is a critical policy question. Some evidence indicates that patients who use opioids will, over time, substitute opioids with medical marijuana (Boehnke et al. 2016; Piper et al. 2017). However, more recent evidence revealed no meaningful decrease in receipt of an opioid in the first 3-months after implementing legalized medical marijuana as compared to states that did not legalize medical marijuana (McGinty et al. 2023). Because our study was cross-sectional and we modeled lifetime marijuana use, we are unable to determine whether prescription opioid use changed after using medical or recreational marijuana. We found no evidence that opioid dose was associated with lifetime marijuana use, but daily vs. less frequent prescription opioid use was positively associated with recreational marijuana use relative to no marijuana use. Though speculative, daily opioid users may be seeking marijuana as an adjunct to opioid therapy, but we expected to find daily opioid users to be primary medical marijuana users. Instead, we observed more frequent opioid users to be more likely recreational marijuana, but not medical marijuana users. This could be explained by the relatively short period of time that medical marijuana has been available in the state of Missouri, although medical marijuana was legal in both states throughout the duration of the study.

Because more frequent opioid use is associated with use of other substances (Hudgins et al. 2019; Rhee and Rosenheck 2021), we expected both medical and recreational marijuana users to also be daily prescription opioid users. Yet, after controlling for confounding, recreational, but not medical marijuana remained significantly associated with daily opioid use. This suggests that daily opioid users are not seeking out prescriptions for medical marijuana despite prior evidence that medical marijuana use is associated with decreased opioid use (Boehnke et al. 2016; Piper et al. 2017). However, these existing studies recruited patients who were customers of a medical marijuana dispensary which limits direct comparison with the current results.

Although speculative, our findings provide some indication that past marijuana use may be an indicator of risk for becoming a daily opioid user. Given more frequent prescription opioid use is linked to increased risk for depression (Scherrer et al. 2022) and use of other substances (Kessler et al. 2001; Prins et al. 2016), providers may consider discussing patients’ recreational and medical cannabis use history as part of identifying candidates for opioid therapy in the context of safer opioid prescribing.

### Limitations

This study should be interpreted in light of several limitations. First, we measured lifetime marijuana use which precludes drawing conclusions regarding the temporal relationship between marijuana and opioid use. Because this analysis uses the baseline data from a larger prospective cohort study on mental health outcomes in chronic prescription opioid use, we had limited survey time to measure marijuana use. Questions used to measure marijuana use were simplistic in that we did not question patients about having a medical marijuana card and we did not ask about other reasons for medical marijuana use. We also defined recreational marijuana use based on reporting use to get high or to feel good. These items were not drawn from existing interviews but were written in the style of structured, diagnostic lay administered mental health surveys. Our questions were designed to identify marijuana use for use as a covariate in analyses of chronic prescription opioid use and risk for new mental health conditions. However, given the rapid rise in access to medical marijuana in the past 3 years, we believed it appropriate to investigate how lifetime marijuana use (medical and recreational) relates to current daily, higher dose prescription opioid use. Inaccurate recollection and social desirability could have influenced responses to survey items. Participants may have relocated throughout their lifetimes resulting in varying access to legalized marijuana. Participants were recruited from two large health care systems in large, midwestern cities and results may not generalize to other regions.

## Conclusions

Recreational, but not medical, marijuana use was associated with increased odds of being a daily, prescription opioid user. Screening for recreational marijuana use among pain patients receiving prescription opioids may be a pathway to patient-provider discussions about the effectiveness of medical marijuana for pain. Screening for marijuana use may identify patients at risk for daily prescription opioid consumption. Further research is needed to determine the long-term pain, opioid and psychosocial outcomes among persons who use marijuana for pain and/or recreation.

## Data Availability

All data generated or analyzed during this study are included in this published article, and its supplementary information files.
